# The Impact of One Carbon Metabolism on Histone Methylation

**DOI:** 10.3389/fgene.2019.00764

**Published:** 2019-08-28

**Authors:** Magdalini Serefidou, Anuroop Venkateswaran Venkatasubramani, Axel Imhof

**Affiliations:** Biomedical Center Munich, Department of Molecular Biology, Ludwig-Maximilians-University Munich, Munich, Germany

**Keywords:** folate, s-adenosylmethionine, histone methlyation, one-carbon metabolism, methioine

## Abstract

The effect of one carbon metabolism on DNA methylation has been well described, bridging nutrition, metabolism, and epigenetics. This modification is mediated by the metabolite S-adenosyl methionine (SAM), which is also the methyl-donating substrate of histone methyltransferases. Therefore, SAM levels that are influenced by several nutrients, enzymes, and metabolic cofactors also have a potential impact on histone methylation. Although this modification plays a major role in chromatin accessibility and subsequently in gene expression in healthy or diseased states, its role in translating nutritional changes in chromatin structure has not been extensively studied. Here, we aim to review the literature of known mechanistic links between histone methylation and the central one carbon metabolism.

## Introduction

In the last decade, the connection between one carbon metabolism and DNA methylation in embryonic development, cancer, and neurodegenerative diseases has been well established (reviewed in (Liu and Ward, 2010; Rush et al., 2014). Such metabolically induced changes in DNA methylation are inherited by offsprings supporting the importance of nutrition in chromatin accessibility within generations (Morgan et al., 1999; Waterland and Jirtle, 2003). As the central factors involved in one carbon metabolism also act as critical cofactors of histone modifiers, it is highly likely that histone methylation is tightly connected to the cell’s metabolic state (Locasale and Cantley, 2011; Lu and Thompson, 2012; Sharma and Rando, 2017). Methylated histones are recognized by the so-called chromatin readers that recruit other molecules resulting in a change of gene activity (Taverna et al., 2007). Methylation of histone 3 (H3) at lysine 4 (K4), K36 or K79 are generally associated with active chromatin, while methylation at K9, K27, and histone 4 (H4) K20 are frequently associated with repressed chromatin. The different methylation sites and methylation degrees are thought to play specific roles at different stages of transcription (Greer and Shi, 2012), making histone methylation a key factor in the regulation of cellular physiology (Sarg et al., 2002; Nottke et al., 2009; Pedersen and Helin, 2010).

Most importantly, SAM levels are influenced by dietary intake, especially in the case of mammals. Nutrients serve as a substrate for one-carbon metabolic pathway, making methylation highly dependent on the availability of certain nutrients. Since the impact of nutrition in DNA methylation is well known, we try to focus and summarize the molecular mechanisms bridging one carbon metabolites and its cofactors with histone methylation.

### One Carbon Metabolism Regulates SAM Levels

Methylation is mediated by the metabolite S-adenosyl methionine (SAM) (Mentch et al., 2015), the synthesis of which is catalyzed from methionine and ATP by methionine adenosyltransferase enzymes (MATs) [or SAM synthetases (SAM-S); will be referred to as MAT] (Cantoni, 1953). MAT enzymes are encoded by the MAT1A and MAT2A genes, where the former is only expressed in the liver while the latter in most mammalian tissues. SAM is used as a methyl-donor by various cytosolic or nuclear methyltransferases, catalyzing the formation of a methylated substrate and S-adenosylhomocysteine (SAH) (Bailey and Gregory, 1999; Tong et al., 2009; Teperino et al., 2010; Mentch et al., 2015). SAH, a potent inhibitor of most known methyltransferases, is hydrolyzed to homocysteine (Hcy) and adenine by S-adenosylhomocysteine hydrolase (AHCY) to prevent its accumulation (Obeid and Herrmann, 2009; Tehlivets et al., 2013). As the increased concentration of Hcy can also have a negative effect on many methyltransferases, it is efficiently remethylated back to methionine in a folate- and/or choline-dependent manner (Yudkoff, 2012).

The folate derivative N-5,10-methylenetetrahydrofolate is synthesized by C-1-tetrahydrofolatesynthase (MTHFD1) and serine hydroxy methyltransferase (SHMT2) (MacFarlane et al., 2009). In serum, it is converted to 5-methyltetrahydrofolate, the predominant form of folate. Ultimately, a cobalamin (vitamin B12)-dependent methionine synthase (MTR or MS) catalyzes the transfer of one methyl group from 5-methyltetrahydrofolate to Hcy. During the choline-dependent remethylation of methionine, choline is oxidized to betaine, which can also provide one methyl group to Hcy. This latter reaction is catalyzed by the enzyme betaine-homocysteine methyltransferase (BHMT), localized only in the mitochondria of liver and kidney (Blom and Smulders, 2011; Muskiet et al., 2018). The remaining 50% of Hcy, which is not converted to methionine, enters the transsulfuration pathway resulting in the generation of cysteine (Škovierová et al., 2016). The first reaction of this pathway is the condensation of Hcy and serine (Ser) to cystathionine that is further hydrolyzed to cysteine and 2-oxobutyrate. 2-Oxobutyrate is decarboxylated to propionyl-CoA and converted to succinyl-CoA, an intermediate of the TCA cycle.

In addition to the regulation of enzymes involved in remethylation of methionine and SAM synthesis, intracellular SAM concentration can also be regulated by SAM metabolizing enzymes such as glycine N-methyltransferase (GNMT), which catalyzes the transfer of a methyl group from SAM to glycine to form sarcosine (Luka et al., 2009). Interestingly, N-5-methyltetrahydrofolate has also been shown to inhibit GNMT (Wang et al., 2011a). Although the binding between GNMT and N-5-methyltetrahydrofolate has been well characterized in the literature, the impact of GNMT expression in methyl-folate-dependent reactions has not yet been elucidated.

In the following section, we will focus on studies involving the components of one carbon metabolism and how nutrient limitation or enzyme disruption affects histone methylation.

### Enzymes and Metabolites of One Carbon Metabolism and Their Regulation of Histone Methylation

MAT (or Sam-S) is arguably the most important enzyme in the methionine-folate cycle. Organisms such as *Saccharomyces pombe* and *Drosophila melanogaster* have a single gene responsible (called sam1 or Sam-S, respectively) for SAM synthesis, making them a simpler model to study the physiological and molecular effects of MAT disruption (Larsson et al., 1996; Hayashi et al., 2018). For instance, genetic ablation of MAT in *S. pombe* decreases SAM levels and the corresponding histone methylation, causing defects in cell growth and cell cycle progression (Hayashi et al., 2018). Similarly, in *S. cerevisae* auxotroph, low methionine concentration results in slower cell division and reduced H3K4 di- and trimethylation. This effect is irrespective of the growth rate or the methyltransferase activity of the enzyme. This observation was also extended to human K562 cells, where both H3K9me3 and H3K4me3 levels are affected (Sadhu et al., 2013). In *D. melanogaster*, the effects vary from developmental regulation, temperature-sensitive sterility in females to changes in position-effect variegation (Larsson et al., 1996). In mammalian cells, MATIIα reduction leads to a derepression of the COX2 gene, which is generally repressed *via* SETDB1-mediated H3K9me3 methylation. MATIIα reduction also results in an overall decrease of H3K4me3 that potentially leads to a general loss in transcriptional activity (Kera et al., 2013). In addition, MATIIα was also found to act as a corepressor of the MafK transcription factor to maintain the repressed state of the HO-1 gene (Katoh et al., 2011). A relatively recent study observed similar effects on HCT116 cells upon methionine restriction and MATIIα knockdown. In both cases, global histone methylation levels are reduced, whereas the levels of H3K9 and K27 mono-methylation remains unchanged (Haws and Denu, 2017). Furthermore, methionine restriction (MR) was shown to have a direct impact on both SAM and H3K4 methylation (Mentch et al., 2015). This was indeed observed upon MATIIα disruption in mammalian cells and *S. pombe* (Kera et al., 2013; Hayashi et al., 2018). Methionine refeeding leads to a reestablishment of the H3K4me levels and similar effects have been observed in a more recent study as well (Dai et al., 2018).

Another SAM-buffering enzyme, which has been underexplored in the context of histone methylation, is GNMT. Increase in GNMT expression causes SAM depletion and sarcosine (N-methylglycine) induction (Obata et al., 2014). This can result in changes of histone methylation status, although such studies have been negligible. Interestingly, GNMT was found to be one of the many folate-binding proteins of the liver and was structurally proven to bind 5-methyl-THF pentaglutamate that inhibits its methyltransferase activity (Cook and Wagner, 1984; Luka et al., 2007). Furthermore, GNMT induction stimulates the remethylation of Hcy by improving folate retention, while the absence of GNMT reduces hepatic folate levels and MTR expression (Wang et al., 2011a). GNMT can also regulate the transsulfuration pathway of Hcy, inducing an increase in cysteine and removal of excess Hcy under high methionine condition (Wang et al., 2011b). In addition, a recent study described a reduced gluconeogenesis in GNMT knockout mice due to a complex metabolic reprogramming towards pathways that utilize elevated SAM levels (Hughey et al., 2018).

Folate is another important metabolite in the one carbon cycle. As mentioned in the previous section, apart from its well-documented role in DNA methylation (Crider et al., 2012; Ly et al., 2012), studies on its impact on histone methylation are sparse. Like most intermediary metabolites, folate can be observed in the nucleus (Zamierowski and Wagner, 1977), where it has been shown to bind and regulate a number of enzymes. One of them is LSD1, which demethylates H3K4me1/2. The importance of folate binding has been suggested to protect LSD1 from inhibition by formaldehyde, a by-product of the demethylase reaction (Luka et al., 2011). This observation was supported by a crystal structure showing the binding of folate in proximity to LSD1’s active site (Luka et al., 2014). In liver samples of mouse fed with a folate-deficient diet, H3K4me is substantially increased, suggesting a reduced activity of LSD1 (Garcia et al., 2016). Moreover, rats fed with folate-deficient diet show a three-fold lower SAM concentration in the liver compared to controls, and a resupplementation of folate positively correlates with SAM concentration (Miller et al., 1994). On the other hand, in a *S. cerevisae* auxotroph strain of folate (*fol3Δ*), SAM levels do not change between wild-type and low folate cultures, but a prominent increase was indeed observed in high folate conditions (Sadhu et al., 2013). This suggests the possibility of organism-specific changes in histone methylation under low folate conditions or the regulation of SAM *via* a methionine or an SAM-independent mechanism. Nevertheless, lower folate level leads to a decrease in H3K4me2/3 and consequently affects gene expression. Interestingly, H3K79me was found to be more resilient than H3K4me. The reasons for this are yet to be explored but may be due to differences in *K*_m_ of the respective methyltransferases for SAM (Sadhu et al., 2013). Surprisingly, basic enzymatic parameters for most histone methyltransferases are not known or vary substantially among different laboratories (Eskeland et al., 2004; Patnaik et al., 2004). Another possible cause for the different susceptibilities of methylation sites is an allosteric regulation of enzymes by additional modifications on the same or proximal nucleosome. Examples for this are the influence of H2B ubiquitination on H3K4 and K79 methylation (Soares and Buratowski, 2013) and the induction of PRC2 activity by the recognition of a preexisting H3K27 methylation (Margueron et al., 2009).

Reports suggest that nutritional folate reduction also results in an increase in the level of plasma Hcy (pHcy) in rats (Miller et al., 1994). Indeed, rats exposed to diets of high methionine, B vitamin deficient, or both show increased plasma Hcy levels (Esse et al., 2013). These diets also induce an increase in SAH levels in the liver, a reduction in H3 arginine 8 dimethylation (H3R8me2) in the brain, and upregulated EZH2 expression and subsequent H3K27me3 at the CFTR promoter (Yang et al., 2018).

The link between Hcy, folate, and SAM levels is through MTHFR that converts Hcy to methionine and maintains intracellular SAM concentration. Indeed, MTHFR has been shown to be an important regulator of heterochromatin maintenance. A knockdown of MTHFR in HeLa cells causes a decrease in H3K9me3 and an increase in centromeric satellite repeat expression (Xiahou et al., 2014). In a subsequent study, cells depleted of MTHFR accumulate in S-phase due to decreased H4K20me3 levels. This, however, is rescued by overexpressing the H4K20 trimethylase SUV4-20H2 (Zhang et al., 2003; Li et al., 2017). Whether this effect is directly due to a reduction in SAM level is questionable, since depletion of SAM by MATIIα/β knockdown results in a G1 arrest by prevention of S phase entry (Lin et al., 2014). On the other hand, serine provides methyl groups for the conversion of THF to methylene-THF that serves as a substrate for MTHFR. The enzyme SHMT accomplishes the conversion of serine to glycine, and it has been shown in yeast (Shm2) to occur *via* a complex called SESAME, along with PHGDH (Ser32), SAM-synthetases, pyruvate kinase (Pyk), and acetyl-CoA synthetase. This complex interacts with Set1, an H3K4 methyltransferase, and regulates H3K4me3 in the promoter of Pyk leading to its subsequent autoregulation (Li et al., 2015).

Hcy remethylation can also be accomplished *via* betaine that is obtained from choline, as catalyzed by the betaine homocysteine S-methyltransferase (BHMT). Dietary choline supplementation in rats was shown to have a strong effect on fetal histone methylation. Specifically, H3K9me2/3 and H3K4me2 are directly and indirectly proportional to choline intake, respectively. Choline intake also downregulates G9a and SUV39H1 (both H3K9 methyltransferases), suggesting that the presence of choline might result in a transcriptionally open environment (Davison et al., 2009). Upon choline deprivation of mouse neural-progenitor cells, H3K9me2 is modified at specific genomic locus, causing poor recruitment of G9a (Mehedint et al., 2010). On the contrary, betaine shows a positive correlation with H3K4me3 level in neuroblastoma cells. The corresponding enzyme, BHMT, believed to be present primarily in adult kidneys and liver, was also found to be expressed in adult central nervous system (CNS), suggesting that betaine can serve as a source of methyl group for Hcy remethylation in other tissues as well (Singhal et al., 2015). A summary of enzymes/metabolites and their impact on histone modifications is given in **Table 1**.

**Table 1:** Overview of enzymes and metabolites and their corresponding roles in histone methylation.

	Enzymes		
	Model system	Subcellular localization	Effect of loss of function on histone methylation
MAT	Mouse embryonic fibroblasts cells: MEFs	Nucleus (MATI/III)	Reduction in H3K4me2/3 and H3K9me3 (Kera et al., 2013)
		Cytoplasm and nucleoplasm (MATII)	
MS	*Drosophila* Schneider cell line 2 (S2)	Cytoplasm, nucleus (*C. albicans*)	Unknown; possible reduction in H3K4me2 (Liu et al., 2015)
	*Drosophila melanogaster* flies		
GNMT	*Drosophila melanogaster* flies	Cytoplasm	Unknown; possible indirect effect *via* buffering SAM levels (Obata et al., 2014)
	Human chondrosarcoma cells: CH2879 H1299		Possible reduction in H3K27me3 *via* EZH2 and H3K9me3
ACHY	Human lung epithelial carcinoma cells: H460, A549, MCF7 and T24	Cytoplasm	*via* SETDB1 and H4K20me3 (based on drug inhibition of the
	Human keratinocyte cells: HaCaT		enzyme (꼴Miranda et al., 2009; Lee and Kim, 2013; Girard et al., 2014)
BHMT	Human postmortem frozen brain tissues	Cytoplasm	Possible reduction in H3K4me3 (Singhal et al., 2015).
	Human neuroblastoma cells: SH-SY5Y		
MTHFD	Arabidopsis plants	Cytoplasm	Possible reduction in H3K9me2
MTHFR	Human cancer cells: HEK293T, HeLa, A549, HT29, and U2OS	Cytoplasm	Reduction in H3K9me3 (Xiahou et al., 2014) and H4K20me3 (Li et al., 2017)
SHMT	Yeast cerevisiae	Nucleus, mitochondria and cytoplasm	Possible reduction in H3K4me3 (Li et al., 2015)
	Metabolites		
Methionine	Human colorectal cancer cells: HCT116 cells	Cytoplasm and mitochondria	Reduction in H3K4me (Mentch et al., 2015; Dai et al., 2018)
	C57BL/6J mice		
Choline	Sprague–Dawley rats	Nucleus, mitochondria, and cytoplasm	Reduction in H3K9me2/3 and increase in H3K4me2 (Davison et al., 2009)
Betaine	Human postmortem frozen brain tissues	Cytoplasm and mitochondria	Possible reduction in H3K4me3 (Singhal et al., 2015).
	Human neuroblastoma cells: SH-SY5Y		
Homocysteine	Wistar rat, C57BL/6J mouse	Cytoplasm	Reduction in H3R8me2 and H3K27me3 (Esse et al., 2013; Yang et al., 2018)
	Human hepatocyte cells: HL-7702		
Cobalamin	–	Cell membrane	Unknown
Serine	Yeast cerevisiae	Cytoplasm and mitochondria	Possible reduction in H3K4me3 (Li et al., 2015)
Folate	C57BL6/J mouse	Nucleus, mitochondria, and cytoplasm	Increase in H3K4me (꼴Garcia et al., 2016)

Histone methylation status can also be influenced by levels of vitamin B_12_, MTHFD1, MTR, and ACHY/SAHH. Although their role in DNA methylation has been documented in some cases, very few studies have been carried out to explicitly investigate their impact on histone methylation.

## Conclusion and Future Perspectives

Studies on transmethylation and one carbon cycle have so far been investigated in the context of DNA methylation. However, in recent years, their impact on histone posttranslational modifications is becoming more and more evident. For instance, the role of GNMT in transmethylation/folate pathway is quite known, but it is surprising that its role in histone methylation has not yet been characterized, although their influence on SAM levels is well established. Moreover, through the roles of Hcy in transsulfuration and GNMT in reprogramming liver metabolism, folate–methionine cycle can now be evidently linked to two important metabolic substrates of posttranslational modifications, SAM, and acetyl-CoA. Studies on the interplay of these two key metabolites and their corresponding changes in histone tail modification would further improve our understanding of the connection between nutrition, metabolism, and epigenetics.

In the last decade, mass spectrometry has offered a more accurate large-scale quantitative analysis of histone modifications than the traditionally used Western blot (Völker-Albert et al., 2018). A new mass spectrometry method for histone posttranslational modification (PTM) analysis provides the possibility of high throughput screening of 200 PTMs in less than a minute (Sidoli et al., 2019a). Furthermore, methods that combine metabolomics and histone PTM analysis have already been developed for acetyl-CoA and histone acetylation (Sidoli et al., 2019b). These developments may not only help to broaden our view of how metabolism and epigenetic processes are connected but also open new pathways for pharmacological intervention in diseases.

Indeed, a surge in systematic effort to develop cancer drugs, inhibitors of methionine, and folate pathway enzymes has been observed in the last years. For instance, 3-deazaneplanocin A (DZNep), an inhibitor of ACHY, regulates the expression of both EZH2, a H3K27 trimethylase, and SETDB1, a H3K9 trimethylase. This drug has been shown to reduce H3K9me2 in human lung cancer cells and H3K27me2 in chondrosarcomas (Miranda et al., 2009; Lee and Kim, 2013; Girard et al., 2014), in addition to reactivating PRC2 repressed genes and inducing apoptosis in cancer cells *via* FBXO32 (Tan et al., 2007). Similar drugs that inhibit MTHFD1, such as LY345899, have been proposed to be potent drugs for anticancer therapy (Gustafsson, 2017). Interestingly, Temozolomide (TMZ), a drug that has been used to induce DNA methylation and has anticancer properties, was found to affect methylation of H3K4 *in vitro* (Wang et al., 2016). More recently, a MAT2A inhibitor known as AG-270 has been used for the treatment of patients with solid tumors or lymphoma in phase 1 clinical trials (Agios pharmaceuticals, Inc., 2018). Whether the effect of these drugs on histone methylation is an important aspect of their function or is only a bystander effect is yet to be determined.

## Author Contributions

MS, AV, and AI participated in drafting the article and revising it critically for important intellectual content; and gave final approval of the version to be submitted. The corresponding author takes primary responsibility for communication with the journal and editorial office during the submission process, throughout peer review, and during publication.

## Funding

This work has received funding from the European Union's Horizon 2020 research and innovation programme under the Marie Skłodowska-Curie grant agreement No 675610; QBM and SFB1309

## Conflict of Interest Statement

The authors declare that the research was conducted in the absence of any commercial or financial relationships that could be construed as a potential conflict of interest.
